# Mitochondrial homeostasis and aging: the mtDNA-cGAS-STING axis

**DOI:** 10.3389/fimmu.2026.1917296

**Published:** 2026-08-25

**Authors:** Ling Wang, Zhengwei Zhang, Binbin Xia, Jun Li, Hongmei You

**Affiliations:** 1 Department of Pharmacy, Shangyu People’s Hospital of Shaoxing, Shaoxing, China; 2 Department of Neurosurgery, Shangyu People’s Hospital of Shaoxing, Shaoxing, China; 3 Inflammation and Immune Mediated Diseases Laboratory of Anhui Province, Anhui Institute of Innovative Drugs, School of Pharmacy, Anhui Medical University, Hefei, China; 4 Department of Pharmacy, Hangzhou Women’s Hospital, Hangzhou, China

**Keywords:** aging, cGAS-STING, immunity, mitochondria, mtDNA

## Abstract

Aging and its associated diseases have become an increasingly severe global health challenge, not only significantly exacerbating the global disease burden but also posing a continuous threat to public health systems worldwide. During the aging process, the aberrant release of endogenous mitochondrial DNA (mtDNA) is a key trigger for the activation of the cGAS–STING innate immune pathway. Existing research has confirmed that the overactivation of the cGAS–STING pathway is the core molecular mechanism driving the senescence-associated secretory phenotype (SASP), chronic inflammation, and organ functional decline. Notably, the mechanisms of mtDNA release and the activation characteristics of the cGAS–STING pathway exhibit significant organ-specificity. Different tissues mediate mtDNA leakage through specific pathways, such as mitochondrial permeability transition, oxidative damage, and defective mitophagy, thereby differentially regulating downstream inflammatory signals. Given the central driving role of the aberrantly activated mtDNA-cGAS-STING axis in age-related organ damage, targeting this pathway has emerged as a promising therapeutic strategy for the systemic mitigation of aging-associated chronic inflammation. This review systematically elucidates the molecular basis of the mtDNA-cGAS-STING pathway, delves into its organ-specific activation mechanisms, and critically evaluates current intervention frameworks and clinical prospects, aiming to provide a theoretical basis and innovative perspectives for the precision prevention and clinical management of age-related diseases.

## 1 Introduction

“Inflamm-aging” is one of the core hypotheses in aging research, referring to a chronic, low-grade inflammatory state that develops with increasing age. The latest research indicates that mtDNA release due to mitochondrial dysfunction and the subsequent activation of innate immunity are key drivers of this process (1). Studies point out that mitochondrial damage, failure of autophagy and mitochondrial quality control mechanisms, and metabolic stress (such as impaired pyrimidine synthesis) are the main causes of abnormal mtDNA leakage into the cytoplasm (2). Cytosolic mtDNA can be sensed by pattern recognition receptors like the cGAS-STING axis or TLR9, triggering the production of type I interferons (IFN-I) and pro-inflammatory cytokines (3, 4). This mtDNA-mediated aberrant immune activation plays a crucial role in the pathogenesis of neurodegenerative diseases (4), skeletal muscle aging (5), osteoporosis (6), and cardiovascular diseases (7). As a key component of the innate immune system, the cGAS-STING pathway initially gained prominence for its core function in combating microbial infections by recognizing pathogen-derived DNA and activating interferon responses (8, 9). With further research, its critical role in aging and age-related diseases has been established. The function of the cGAS-STING pathway in the aging process is complex; it is not only a primary mechanism mediating chronic inflammation and the SASP but also plays an important role in maintaining tissue homeostasis and organismal lifespan (10, 11). However, the homeostatic regulatory function of this process is highly dependent on biological context and activation thresholds (12). During aging, chronic mitochondrial stress and persistent mitochondrial DNA accumulation drive a progressive shift in the cGAS-STING signaling threshold, transforming it from a transient, protective immune-surveillance state into a chronic, pathological activation state. This aberrant and sustained overactivation serves as a core driver of “inflamm-aging,” ultimately accelerating organ functional decline, tissue fibrosis, and the development of various age-related diseases (13). Currently, related research has broadly covered fields such as neurodegenerative diseases (e.g., Alzheimer’s disease (10), Parkinson’s disease (14), and amyotrophic lateral sclerosis (15), cardiorenal aging (16), chronic inflammation, and immunosenescence (11). Existing evidence shows that cytosolic DNA (including damaged mitochondrial DNA, micronuclear DNA, and chromatin fragments) can activate the cGAS-STING pathway. Within the specific context of aging and cellular senescence, mtDNA has emerged as a unique and predominant class of damage-associated molecular patterns (DAMPs). Unlike nuclear-derived DNA fragments that typically result from genomic instability or cellular crisis, the aberrant release of mtDNA reflects a distinct “mitochondria-immune signaling” transduction mechanism, which constitutes the core focus of this review. Although the pathway’s role in the aging of various organs such as the heart, kidneys, and brain has been confirmed, it is not yet clear how different tissues form specific “immune fingerprints.” In light of this, this study integrates evidence from multiple organs, including the heart, kidneys, and brain, to deeply elucidate the dynamic balance between mitochondrial quality control and inflammatory signaling, thereby constructing a unified theoretical framework for understanding cross-organ aging mechanisms.

## 2 The molecular basis of the mtDNA-cGAS-STING pathway

### 2.1 Mechanisms of mtDNA release

During the aging process, the cytosolic release of mtDNA is a complex pathophysiological process driven by the synergy of multiple factors, with mitochondrial dysfunction and loss of structural integrity at its core (17, 18). Based on current research, the key triggering mechanisms for this process primarily include: oxidative stress-mediated mitochondrial damage, increased mitochondrial outer membrane permeabilization (MOMP), and the functional decline of quality control systems such as mitophagy (19, 20).

Oxidative stress is widely recognized as an initiating factor. In various age-related pathological models, the excessive accumulation of reactive oxygen species (ROS) directly damages and fragments mtDNA, creating the conditions for its subsequent release (21, 22). For example, in an Alzheimer’s disease model, Aβ-induced oxidative stress leads to the oxidation, fragmentation, and release of mtDNA, which is subsequently recognized by ZBP1 (21). Unlike cGAS-STING, which primarily triggers an IFN-driven SASP, ZBP1-mediated sensing often promotes programmed necrosis or inflammasome activation. These parallel sensing mechanisms function in concert to fine-tune the intensity and character of inflammatory responses within senescent tissues (23, 24). In an acute pancreatitis model, excessive mitochondrial ROS similarly disrupts mitochondrial integrity, causing mtDNA leakage (22). The direct pathway for mtDNA release depends on the formation of pores on the mitochondrial membrane. Studies have confirmed that the activation of pro-apoptotic proteins BAX and BAK, or the oligomerization of the mitochondrial outer membrane protein voltage-dependent anion channels 1(VDAC1), can form pores in the outer mitochondrial membrane, mediating the release of mtDNA (25, 26). Cytosolic free BAX and BAK proteins aggregate on the outer mitochondrial membrane, forming large, ring-like porous structures (macropores). Due to the large diameter of these pores, the inner mitochondrial membrane (IMM), along with its enclosed matrix components (including mtDNA), undergoes “herniation” through the pores. This physical tension ultimately leads to the rupture of the inner mitochondrial membrane, causing the mtDNA, originally confined within the mitochondrial matrix, to be fully released into the cytoplasm (27). Unlike BAX/BAK-mediated leakage, VDAC1-mediated mtDNA leakage is a non-apoptotic mechanism. In this mechanism, VDAC1 proteins on the outer mitochondrial membrane oligomerize under stress to form channels and, through engagement with the opening of the inner membrane’s mitochondrial permeability transition pore (mPTP), release mtDNA from the matrix into the cytoplasm (28). Mitochondrial Transcription Factor A (TFAM), a critical dual-function protein, not only participates in mtDNA replication and transcription but also acts like a histone, compacting mtDNA into stable “nucleoid” structures. Under conditions of oxidative stress, DNA damage, or metabolic stress, mitochondrial homeostasis is compromised, and VDAC proteins oligomerize to form sufficiently large channels. The TFAM protein, tightly bound to mtDNA, can then leak through these channels and be released into the cytoplasm (29, 30).

Concurrently, the failure of the mitochondrial quality control system, particularly the decline in mitophagy efficiency, is another key factor that exacerbates mtDNA release (17, 31). Under physiological conditions, autophagy can identify and clear damaged mitochondria. When autophagy is dysfunctional, the ubiquitination marking of damaged mitochondria and their recognition by autophagy receptors are obstructed, leading to the accumulation of a large number of dysfunctional mitochondria that cannot be cleared in a timely manner. These dysfunctional mitochondria continuously produce excessive ROS, which can directly attack mtDNA, causing strand breaks and base oxidation, thereby compromising its molecular stability and increasing the risk of its release into the cytoplasm. Furthermore, under the influence of multiple stress factors such as calcium homeostasis imbalance, high levels of ROS, and low ATP, these long-retained damaged mitochondria are prone to inducing the persistent opening of the mPTP. The opening of the mPTP causes a sharp increase in inner mitochondrial membrane permeability, leading to a massive influx of ions and hyperosmotic swelling of the matrix. Due to the limited elasticity of the outer mitochondrial membrane, the extreme swelling of the inner membrane eventually causes the outer membrane to rupture, resulting in the passive and massive release of mtDNA, originally located in the mitochondrial matrix, into the cytoplasm (1, 32). For example, in a cardiac hypertrophy model, PINK1-mediated mitophagy can alleviate the pathological process by suppressing the cGAS-STING pathway activated by mtDNA release (33), whereas Thymic Stromal Lymphopoietin (TSLP) exacerbates mtDNA release by inhibiting mitophagy (34). Interestingly, the latest research has found that released TFAM can also act as a receptor, binding to the LC3 protein and helping the cell clear the leaked mtDNA through the autophagy pathway. This further reveals the complexity of TFAM’s role in cellular stress responses.

Ultimately, the mtDNA leaked into the cytoplasm (especially in its oxidized form) acts as a damage-associated molecular pattern (DAMP), recognized by innate immune pathways such as cGAS-STING, triggering a persistent inflammatory response and cellular senescence program, forming a vicious cycle that amplifies and accelerates the aging process (35, 36).

It is important to note that BAX/BAK-mediated pore formation is dose- and context-dependent (37). While massive activation leads to rapid apoptosis, sub-lethal or sustained low-level activation of these pores allows for the selective release of mtDNA without triggering the full-fledged caspase-dependent apoptotic cascade. This ‘leaky’ mitochondrial state enables the persistence of aging cells, which harbor damaged mitochondria and continuously secrete SASP factors, rather than undergoing cell death (25).

### 2.2 Activation of the cGAS-STING signaling pathway and SASP formation

The cGAS-STING signaling pathway is a core component of the mammalian innate immune system, with its primary function being to monitor and respond to abnormally present DNA in the cytoplasm (38). Under physiological conditions, DNA is primarily confined to the nucleus and mitochondria; its appearance in the cytoplasm typically signifies pathogen invasion or host cell damage and is therefore highly immunogenic (38). The pathway recognizes both exogenous DNA from pathogens (PAMPs) and DAMPs, using them as signals to initiate a robust inflammatory response (10). Although the cGAS-STING pathway was initially identified as a critical defense line against pathogen infection, recent research has revealed its pivotal roles in numerous non-infectious physiological and pathological processes, including the development of autoimmune diseases, regulation of anti-tumor immunity, cellular senescence, and tissue damage repair. These discoveries have significantly broadened our understanding of the pathway’s role in both maintaining human health and driving disease progression (39).

As a cytosolic DNA sensor, cGAS recognizes abnormally localized dsDNA in the cytosol, which can originate from pathogen invasion or the host itself (e.g., mitochondrial DNA, extranuclear chromatin). Upon recognition, cGAS is activated and catalyzes the synthesis of the second messenger 2′,3′-cyclic guanosine monophosphate-adenosine monophosphate (2′,3′-cGAMP) (40, 41). cGAMP subsequently binds to and specifically activates the adaptor protein STING, which is anchored to the endoplasmic reticulum (ER) (42). In its resting state, STING (apo-STING) is sequestered in the ER in an auto-inhibited conformation, which prevents TBK1 recruitment (43). Upon binding the second messenger cGAMP, STING undergoes a conformational shift that triggers its translocation from the ER to the Golgi apparatus, where it functions as a scaffold for TBK1 recruitment and activation (43). Following transport, the activated STING recruits and activates TBK1, which in turn mediates the phosphorylation and nuclear translocation of IRF3. Concurrently, the NF-κB signaling pathway is also activated, and these two pathways ultimately work in concert to induce the expression of type I interferons (IFN-α/β) and other pro-inflammatory cytokines (44).

**Figure 1:**
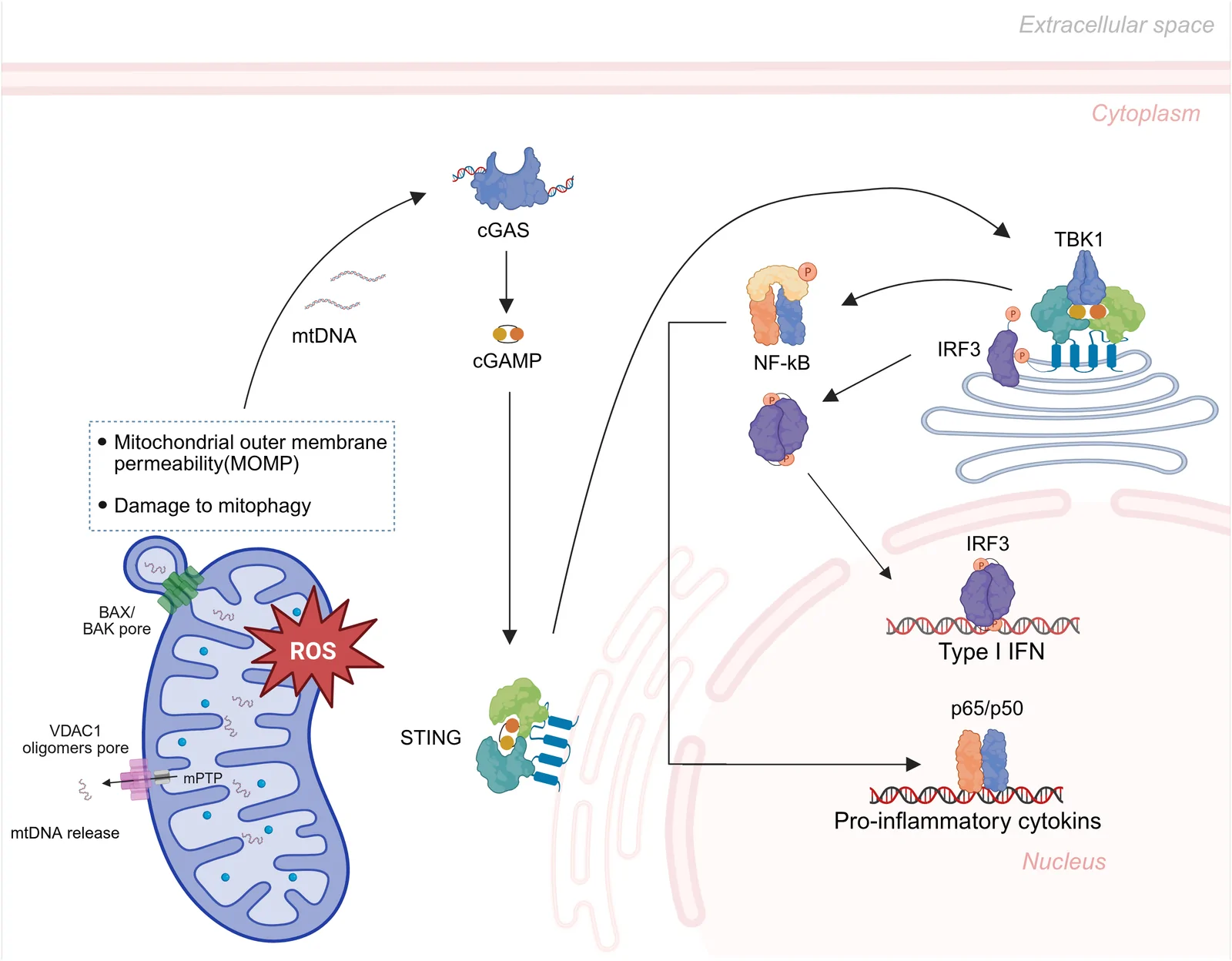
The mtDNA–cGAS–STING signaling axis and pathological SASP formation. Mitochondrial stressors, including ROS and metabolic imbalances, promote mitochondrial outer membrane permeability through pore-forming proteins such as BAX/BAK and VDAC1, as well as mPTP opening, thereby facilitating mtDNA release. Cytosolic mtDNA is sensed by cGAS, which catalyzes the conversion of ATP and GTP to cGAMP. cGAMP then binds to STING at the endoplasmic reticulum, driving STING conformational maturation and translocation to the Golgi apparatus. Activated STING recruits and activates the TBK1-IRF3 axis and the NF--κB pathway, resulting in the secretion of pro-inflammatory SASP factors. Red arrows indicate activation/translocation steps. Abbreviations: MOMP, mitochondrial outer membrane permeabilization; mPTP, mitochondrial permeability transition pore; VDAC1, voltage-dependent anion channel 1; SASP, senescence-associated secretory phenotype.

Activated IRF3 and NF-κB act synergistically, binding to the promoter regions of SASP-associated genes (e.g., IL-6, IL-8, CXCL10, CCL2, IFN-α) to promote the transcription and secretion of pro-inflammatory factors, chemokines, and matrix metalloproteinases. It has been demonstrated in multiple models that knocking down/knocking out cGAS or STING, or using their inhibitors (such as H151, engineered exosomes for targeted delivery of LINE1 antisense oligonucleotides, and hydroxychloroquine), can significantly reduce the phosphorylation levels of p-TBK1, p-IRF3, and p-p65, subsequently inhibiting the expression of SASP factors and ameliorating senescence-associated phenotypes. The role of the cGAS-STING signaling pathway in regulating SASP has been validated in various cell types, including glial cells (45, 46), cardiomyocytes (47), ovarian granulosa cells (48), and vascular endothelial cells (49, 50). The molecular choreography of the mtDNA-cGAS-STING axis is illustrated in **Figure 1**, which delineates the transition from mitochondrial membrane stress—mediated by VDAC1, BAX/BAK, and mPTP pores—to the cytosolic recognition of mtDNA by cGAS. Following the synthesis of cGAMP, **Figure 1** further highlights the STING-dependent recruitment of TBK1 and the subsequent nuclear translocation of IRF3/NF-κB, which sets the transcriptional stage for SASP induction.

## 3 Organ specificity of the mtDNA-cGAS-STING axis

### 3.1 Central nervous system

Studies indicate that in melatonin-deficient mice (AANAT-KO) and Huntington’s disease models, melatonin deficiency or the expression of mutant huntingtin protein induces a decrease in MMP and increases mitochondrial membrane permeability, thereby promoting the release of mtDNA into the cytosol. Cytosolic mtDNA can activate the cGAS-STING-IRF3/NF-κB pathway, driving neuroinflammation and cell death; interventions with exogenous melatonin, mtDNA clearance, or cGAS inhibition can effectively block this cascade, exerting a neuroprotective effect (4). This core mechanism first clarified the pro-inflammatory role of mitochondrial dysfunction in aging and various neurodegenerative diseases, and has been further validated in Alzheimer’s disease (AD). In AD pathology, amyloid-beta (Aβ) and tau protein undergo non-enzymatic glycation to form advanced glycation end-products (AGEs), among which glycated Aβ (gAβ) has an extremely high affinity for the receptor for AGEs (RAGE). The gAβ-RAGE interaction not only induces neuroinflammation but also leads to exacerbated synaptic toxicity. During this process, VDAC1-mediated oligomerization of the outer mitochondrial membrane forms pores, leading to the leakage of mtDNA and other contents into the cytosol, which in turn activates the cGAS-STING pathway within neurons and promotes the progression of AD pathology (51). Zhao et al. (52) proposed that mtDNA released from neurons under mitochondrial oxidative stress can act as a signaling molecule to activate the cGAS-STING pathway in neighboring microglia and astrocytes. This intercellular communication mechanism further drives the polarization of microglia towards the pro-inflammatory M1 phenotype and the transformation of astrocytes into the neurotoxic A1 phenotype, thereby creating a pro-inflammatory microenvironment and exacerbating the disease process. Notably, the cGAS-STING pathway exhibits cell-type-specific heterogeneity within the CNS. Existing evidence suggests that microglia, as the brain’s resident immune cells, possess a robust cGAS-STING signaling repertoire, making them the primary orchestrators of the cytokine storm during neuroinflammation (53). In contrast, while neurons also contain mtDNA, their cGAS-STING axis typically functions at a more restrained threshold to avoid unwarranted cell deat (54, 55). Furthermore, the role of cerebral vascular endothelial cells (ECs) should not be overlooked. Recent studies show that endothelial cGAS-STING activation in response to leaked mtDNA directly undermines blood-brain barrier (BBB) integrity via the downregulation of tight junction proteins. This BBB disruption subsequently creates a recursive feed-forward loop, allowing peripherally derived DAMPs and immune cells to infiltrate the CNS, thereby exacerbating the neurodegenerative microenvironm (55). In summary, although the triggers for mtDNA leakage vary among different neurodegenerative diseases, the underlying mechanism ultimately converges on the common pathway of mitochondrial membrane damage, which in turn drives similar neuroinflammatory cascades via the cGAS-STING axis. While the fundamental role of the mtDNA-cGAS-STING axis in the aging of the nervous system is now recognized, its specific role in shaping Central Nervous System (CNS) aging-related pathological features, such as blood-brain barrier disruption and neuroinflammation, still requires further elucidation. This suggests that research into the mechanisms by which this signaling pathway integrates with and influences the overall functional decline of the CNS remains insufficient.

### 3.2 Liver

Hepatocytes are characterized by a remarkably high density of mitochondria, which is consistent with their high metabolic requirement for maintaining energetic and synthetic homeostasis (56). Due to the lack of histone protection, mtDNA is susceptible to oxidative stress, leading to deletions, point mutations, and increased heteroplasmy, which disrupt the oxidative phosphorylation system and affect energy metabolism and ROS balance (57, 58). In senescent hepatocytes, the oligomerization of VDAC and the opening of the mPTP—triggered, for instance, by acetylated MCU-mediated calcium influx—are key pathways for the cytosolic release of mtDNA (59). In a liver ischemia-reperfusion (IR) model, bone marrow-derived macrophages from aged mice exhibit decreased PINK1/Parkin-mediated mitochondrial ubiquitination and lysosomal biogenesis and dysfunction due to dysregulation of the mTOR/Transcription Factor EB (TFEB) signaling pathway (60). The obstruction of mitophagy prevents the effective clearance of damaged mitochondria, thus increasing the risk of mtDNA leakage into the cytoplasm. Cytosolic mtDNA is recognized by cGAS, activating the downstream STING signaling pathway (61). In aged liver macrophages, the activation levels of STING and its downstream molecule TBK1, stimulated by mtDNA, are significantly elevated (62). The activation of the STING pathway further promotes the activation of the NLRP3 inflammasome, leading to the excessive secretion of multiple pro-inflammatory cytokines and chemokines, including IL-1β and IL-18, which exacerbates sterile inflammation and tissue damage in the liver (62). Furthermore, in the aging liver, the deletion of Ribosomal S6 Kinase 1 (S6K1), a downstream effector of the mTOR pathway, can significantly suppress the SASP. Studies show that S6K1 is crucial for the effective activation of IRF3; even in the presence of upstream stimuli like mtDNA, the absence of S6K1 leads to impaired IRF3 activation. This mechanism ultimately ameliorates tissue inflammation and immune cell infiltration. This research proposes a disruptive idea: senescent cells can be transformed into a “benign” state. By inhibiting S6K1, senescent cells persist, but their harmful pro-inflammatory “alarm” is disarmed, thereby avoiding excessive immune responses and tissue damage. This finding expands anti-aging strategies from the singular approach of “clearing senescent cells” to the new dimension of “remodeling senescent cell function,” opening up new avenues for developing safer and more precise anti-aging therapies (63). However, we believe that the above studies have not addressed the complex intercellular interactions in the aging liver, particularly the lack of in-depth exploration of the involvement of parenchymal and non-parenchymal cells such as hepatic stellate cells. Notably, differing from the macrophage-centric path of the studies above, Gu et al (64). found that in a manganese ion (Mn^2+^)-induced liver fibrosis model, the damage and release of mtDNA can trigger the cGAS-STING signaling pathway in hepatic stellate cells, thereby inducing cellular senescence and the formation of a SASP. This discovery provides new insights for developing therapeutic strategies with dual anti-fibrotic and senolytic effects.

### 3.3 Kidney

With age, mitochondrial function in kidney cells (especially renal tubular cells) becomes dysfunctional (65). This dysfunction leads to the release of mtDNA from mitochondria into the cytoplasm (66). For instance,nucleotide metabolic imbalance leads to mtDNA replication errors, which can induce its release in the kidneys of aging mice, activate the cGAS-STING signal, and induce the SASP (67) (68) (69). This chronic, low-grade inflammatory response (“inflammaging”) is a major driver of renal aging, fibrosis, and functional decline (70). Multiple studies collectively confirm that mitochondrial damage is the upstream trigger for this series of events. For instance, the loss of mitochondrial TFAM or deficiency of PINK1 leads to mitochondrial dysfunction, aberrant mtDNA packaging and release, ultimately activating the cGAS-STING pathway and exacerbating renal inflammation, fibrosis, and senescence-associated phenotypes.

The mitochondrial quality control protein PINK1 plays a critical protective role in the process of renal aging. Studies have shown that 24-month-old PINK1 knockout mice exhibit more severe renal fibrosis and tubular injury compared to wild-type mice of the same age. The loss of PINK1 function leads to the accumulation of dysfunctional mitochondria, which in turn triggers the cGAS-STING innate immune pathway, accelerating renal fibrosis and functional decline by amplifying the SASP (65). Although this study confirmed that PINK1 deletion significantly exacerbates age-related renal phenotypes, the specific downstream molecular mechanisms by which it triggers mtDNA release and cGAS activation remain unclear. Based on existing literature, we postulate that PINK1 deficiency primarily induces the release of mtDNA by inhibiting mitophagy, leading to the accumulation of damaged mitochondria and their dysfunction (e.g., increased reactive oxygen species levels, loss of membrane potential) (65). Other research has pointed out that the aberrant release of mtDNA is closely linked to its impaired packaging mechanism. Aging leads to a decline in TFAM function, significant loss of mitochondria, and disordered mtDNA packaging in renal tubular epithelial cells, which promotes the translocation of mtDNA into the cytosol; subsequently, cytosolic mtDNA induces sterile inflammation and promotes tissue fibrosis by activating the cGAS-STING-NF-κB axis (71). These two studies, approaching from different dimensions of mitochondrial homeostasis, profoundly reveal the mechanisms of “mitochondrial-derived inflammation” in renal aging.

### 3.4 Cardiovascular system

The mtDNA-cGAS-STING pathway plays a critical pro-inflammatory role in the pathophysiology of cardiovascular diseases. When myocardial, vascular, or other related cells experience stress such as metabolic dysregulation, ischemia-reperfusion injury, or pressure overload, mitochondrial function is impaired, leading to the release of mtDNA into the cytoplasm (72, 73) Cytoplasmic mtDNA is recognized by cGAS, which in turn activates the STING pathway, triggering a downstream interferon response and inflammatory cascade that leads to pathological changes such as myocardial fibrosis (74), cardiac dysfunction (75), endothelial dysfunction (76), and vascular senescence (77).

In heart failure, cardiomyocyte-specific deficiency of carnitine acetyltransferase (CRAT) disrupts cholesterol metabolism, resulting in the accumulation of the bile acid intermediate 7-HOCA and subsequent mitochondrial DNA stress. This process triggers cGAS–STING-dependent type I interferon signaling, which upregulates AIM2 expression to prime the AIM2 inflammasome, thereby promoting chronic myocardial inflammation and the development of dilated cardiomyopathy (78). Unlike broadly attributing mitochondrial dysfunction to “energy deficiency,” this study used LC-MS/MS to identify 7-HOCA, defining it as a direct pathogenic “metabolic toxin” within cardiomyocytes and providing an excellent molecular target for the future development of specific metabolic enzyme inhibitors or signaling blockers. In myocardial ischemia/reperfusion (I/R) injury, cardiomyocyte-specific overexpression of Parkin enhances mitophagy, clears damaged mitochondria, and limits the release of cytoplasmic mtDNA, thereby blocking the cGAS-STING axis-mediated inflammatory response and exerting a cardioprotective effect (79). In this study, although PARKIN overexpression significantly reduced infarct size on the first day post-surgery, early improvement in cardiac function was not significant; functional benefits primarily appeared two weeks later. This indicates that the protective mechanism of Parkin lies not only in reducing acute necrosis but, more importantly, in accelerating the recovery process from myocardial “stunning” by inhibiting the subsequent inflammatory response, thus effectively delaying pathological ventricular remodeling. In diabetic cardiomyopathy, lipotoxicity-induced mitochondrial oxidative damage and cytoplasmic mtDNA release activate the cGAS-STING signaling pathway, which then induces cardiomyocyte pyroptosis by activating the NLRP3 inflammasome, ultimately leading to cardiac hypertrophy and dysfunction (80). Although lipotoxicity is known to induce endoplasmic reticulum (ER) stress, which may lead to the translocation of STING from the ER to the Golgi apparatus, this study did not delve into the precise contribution of ER stress to the activation of the cGAS-STING pathway. This suggests a potential “dual-trigger” mechanism, whereas the current study focused only on DNA sensing initiated by mtDNA, failing to reveal whether a synergistic enhancing effect exists between the two. Research in atherosclerosis has further confirmed that mtDNA-induced activation of the cGAS-STING pathway plays a key role in promoting NLRP3-dependent pyroptosis. As an important cytoskeletal scaffold protein, increased expression of IQGAP1 in the high-lipid environment of atherosclerosis promotes ROS production, particularly leading to elevated levels of mitochondrial superoxide (mtROS), which damages the integrity of the MMP and membrane structure. In endothelial cells, IQGAP1 triggers mtDNA release by inducing mitochondrial oxidative stress, which in turn activates the cGAS-STING pathway, inducing NLRP3-dependent pyroptosis in vascular endothelial cells and thereby accelerating the pathological progression of atherosclerosis. Experiments have confirmed that inhibiting IQGAP1 or blocking the cGAS-STING axis can effectively alleviate endothelial cell pyroptosis and atherosclerotic lesions, providing potential therapeutic targets for the prevention and treatment of related cardiovascular diseases (81).

### 3.5 Ocular

With the aging of the population, diseases associated with ocular aging, such as dry eye disease (DED), diabetic retinopathy (DR), age-related macular degeneration (AMD), and meibomian gland dysfunction, have become major causes of vision loss. In recent years, mitochondrial dysfunction has been regarded as a key driver of these diseases, as the leakage of mtDNA can activate the innate immune pathway cGAS-STING, inducing chronic inflammation and cellular senescence phenotypes.

DED is the most common chronic inflammatory disease of the ocular surface, and inflammation is its core pathological basis. Studies have shown that stresses such as hyperosmolarity can lead to the opening of the mPTP in ocular surface epithelial cells, triggering the leakage of mtDNA into the cytoplasm and the activation of cGAS, which in turn triggers a cascade of inflammatory responses (82). Both the mPTP inhibitor cyclosporine A and the STING inhibitor C-176 can significantly alleviate symptoms. By excluding traditional hypotheses such as “oxidatively modified mtDNA” and “BAX-mediated release,” this study confirmed through specific inhibitor control experiments that the physical leakage of intact mtDNA through the mPTP is the primary pathway for cGAS-STING activation, providing a theoretical basis for targeting the mPTP rather than relying on antioxidant therapy (83). It is worth noting that mitochondrial damage is usually accompanied by mitophagy to clear damaged mitochondria. Although mitochondrial dysfunction was observed in this study, it did not further explore whether mitophagy defects were a prerequisite for mtDNA accumulation and leakage.

DR is the leading cause of vision impairment and blindness in the working-age population, and its incidence rises significantly after the age of 60. The retinal pigment epithelium (RPE) constitutes the outer blood-retina barrier and is crucial for maintaining retinal homeostasis. Because it is adjacent to the choroidal capillaries, the RPE is susceptible to the effects of hyperglycemia and inflammatory cytokines (84). In the high-glucose environment of diabetes, the mitochondrial membrane potential of the RPE decreases, and the mPTP opens, leading to the leakage of mtDNA into the cytoplasm and the activation of the cGAS-STING pathway. This subsequently upregulates the expression and release of inflammatory cytokines such as TNF-α and IL-6, disrupts RPE function and tight junction proteins, weakens the blood-retina barrier, and promotes the progression of DR. The study also found that high-glucose conditions inhibit RPE mitophagy, and impaired mitophagy causes the accumulation of damaged mitochondria and leaked mtDNA within the cells, continuously activating cGAS-STING. The use of STING inhibitors can restore RPE function and alleviate these changes (85).

AMD is the primary cause of blindness in people over 55 in developed countries. Non-neovascular (dry) AMD accounts for 80-90% of cases, and if not treated in time, it can lead to permanent vision loss (86). Research indicates that RPE dysfunction is a key factor in the development of dry AMD. A large number of senescent cells have been observed in the RPE layer of elderly patients, suggesting that RPE cell senescence is closely related to AMD. Serum iron levels are slightly elevated in aging populations, and iron accumulation has been found in the eyes of AMD patients, particularly in the RPE and Bruch’s membrane; *in vitro*, ARPE-19 cell death is accompanied by an increase in intracellular iron, ROS, and lipid peroxidation levels. NCOA4 is a selective autophagy receptor that can mediate ferritinophagy, leading to an increase in free iron. The study suggests that NCOA4 is the upstream key regulatory factor that initiates this cascade. Inhibiting NCOA4 or using iron chelators (such as DFO) can block iron overload, protect mitochondrial function, prevent mtDNA leakage, and thereby inhibit cGAS-STING-mediated inflammation and senescence, providing a new therapeutic approach targeting the crosstalk between iron metabolism and immunity (87).

**Figure 2:**
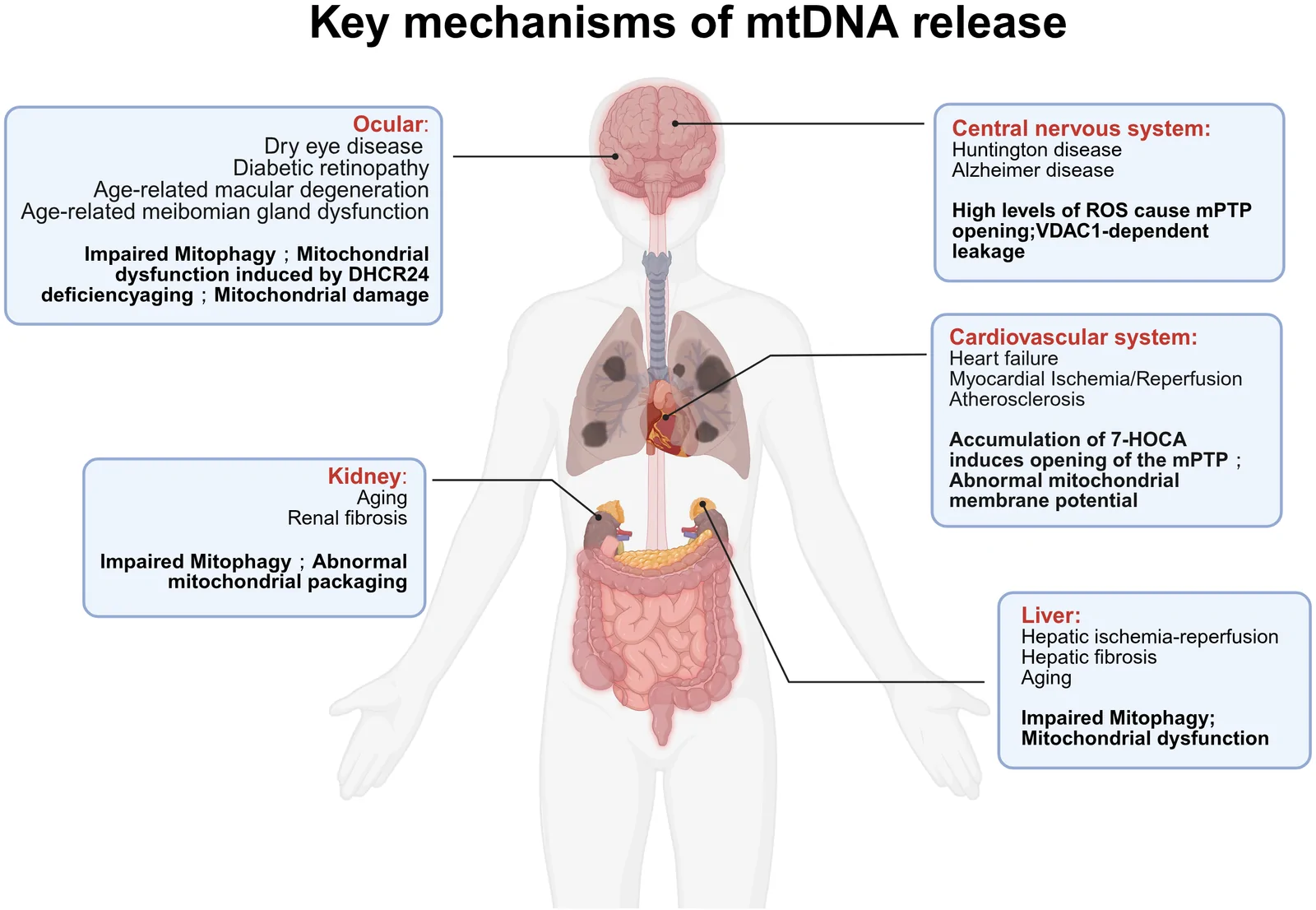
Schematic overview of the role of mtDNA release in age-related diseases. Mitochondrial dysfunction and the subsequent release of mtDNA into the cytosol or extracellular space contribute to the pathogenesis of various clinical conditions. The figure maps these conditions to their respective organ systems, including: (1) Ocular disorders (e.g., dry eye, retinopathy); (2) Neurological diseases (e.g., Alzheimer’s, Huntington’s); (3) Cardiovascular diseases (e.g., heart failure, ischemia/reperfusion); (4) Renal and hepatic pathologies. Associated mechanisms such as defective mitophagy, oxidative stress-induced mPTP opening, and altered mitochondrial membrane potential are summarized for each systemic manifestation.

Age-related meibomian gland dysfunction (ARMGD) is a common ocular surface disease characterized by abnormal lipid secretion and chronic inflammation; it primarily affects the elderly and is a major cause of evaporative dry eye. A study by Cai et al. (88) through multi-omics screening, found that DHCR24, a key enzyme in cholesterol synthesis, is significantly downregulated in aging meibomian glands. Using a meibomian gland-specific DHCR24 knockout mouse model, they confirmed that DHCR24 deficiency leads to lipid metabolic imbalance and mitochondrial dysfunction, subsequently causing mtDNA leakage into the cytoplasm. The leaked mtDNA activates the cGAS-STING signaling pathway, triggering NF-κB-mediated chronic inflammation and cellular senescence and accelerating gland atrophy. Recovering DHCR24 expression via AAV can reverse these pathological changes, indicating that DHCR24 is a key regulatory node connecting lipid metabolic homeostasis with innate immune inflammation (89). However, the specific biophysical mechanism by which cholesterol homeostasis imbalance disrupts mitochondrial membrane stability still requires more refined lipidomics and structural biology evidence. Furthermore the current development of DHCR24 agonists is lagging, which limits the immediate translational application of this discovery. We synthesized the organ-specific pathophysiological mechanisms in **Figure 2**, mapping the transition from tissue-specific mitochondrial stress (e.g., lipid metabolic imbalance in the liver, high-glucose stress in ocular RPE) to the persistent activation of the mtDNA-cGAS-STING immune fingerprint. This figure underscores the convergence of diverse mitochondrial damage signals onto a common inflammatory effector axis across systemic pathologies.

## 4 Therapeutic strategies

Existing research has confirmed that mtDNA plays a pivotal role in the onset and progression of cellular senescence and related diseases. Current research focuses on elucidating how mitochondrial dysfunction triggers cytosolic mtDNA leakage and subsequently induces the SASP and inflammatory responses by activating the cGAS-STING pathway. Consequently, targeting the mtDNA-cGAS-STING pathway has shown immense potential for anti-aging and therapeutic applications for related diseases. Intervention strategies in this field are primarily dedicated to the precise inhibition of the pathological activation of this pathway, which can be categorized into several levels: blocking mtDNA release at the source, and inhibiting the activity of cGAS or STING at key nodes.

### 4.1 Blocking mtDNA release

Firstly, enhancing the mitochondrial quality control system aims to prevent mtDNA release at its source by maintaining mitochondrial homeostasis. This includes enhancing mitophagy to clear damaged mitochondria (60, 88), repairing mtDNA damage (90), and maintaining the balance of mitochondrial biogenesis and dynamics (91). Beyond pharmacological regulation of mitochondrial function (69), the natural compound honokiol (HKL) can also effectively repair mitochondrial damage and block mtDNA leakage by activating the mitochondrial deacetylase Sirt3. In animal models, a single dose of HKL (5 mg/kg) was able to reduce the fibrosis score by about 50% and restore mitochondrial morphology and lung tissue function, thus demonstrating significant anti-fibrotic and anti-aging potential (92). Meanwhile, Mao et al. (93) constructed a nanosystem co-assembled from epigallocatechin gallate (EGCG) and quercetin (Que) (EQ NPs), which can effectively block the inflammatory cascade initiated by mitochondrial-derived signals through multi-modal control of mtDNA levels. EQ NPs have excellent ROS scavenging capabilities, which can inhibit the imbalance of mitochondrial homeostasis at the source. Furthermore, EQ NPs can also directly reduce the production of Ox-mtDNA, prevent the excessive opening of the mPTP, and decrease the initial leakage of mtDNA into the cytoplasm. Once mtDNA escapes into the cytoplasm, EQ NPs can use their polyphenol-rich flavonoid structure on the surface to “capture” it through adsorption, thereby lowering the intracellular mtDNA level. Finally, EQ NPs can also promote mitophagy, clear damaged mitochondria, and effectively prevent them from being recognized as DAMPs by intracellular immune sensors. This study achieved precise blockade of the inflammatory cascade induced by mitochondrial dysfunction through a multi-modal, comprehensive regulatory strategy. Moreover, compared to expensive monoclonal antibodies or growth factors, EQ NPs based on natural polyphenols significantly reduce raw material costs while showing better industrial translation potential and application prospects.

Besides maintaining mitochondrial homeostasis, one can also choose to directly inhibit the mtDNA release channels. This approach focuses on the physical channels through which mtDNA escapes from the mitochondria. This strategy serves as a second line of defense when the mitochondrial quality control system fails or is damaged. Research indicates that targeting mechanisms such as VDAC1 oligomerization may be an effective route. As a key regulatory channel linking mitochondrial stress to cytoplasmic inflammation, VDAC1 is considered a highly promising therapeutic target (35). Given that VDAC1 is an essential protein for cellular metabolism, completely inhibiting its function is not feasible. Therefore, the core of the therapeutic strategy lies in selectively blocking its pathological oligomerization. The small molecule inhibitor VBIT-4 effectively prevents the formation of large pores under stress by stabilizing the monomeric conformation of VDAC1 or interfering with its aggregation, thereby blocking mtDNA penetration. In studies of neutrophils from patients with systemic lupus erythematosus and motor neurons in amyotrophic lateral sclerosis, VBIT-4 has been shown to effectively inhibit the formation of large pores on the outer MOM, blocking the release of mtDNA into the cytoplasm at its source (28). Compared to the severe side effects that direct blockade of the cGAS-STING system may cause, such as impaired host anti-pathogen capabilities, interventions targeting VDAC1 can alleviate inflammation while better maintaining the host’s basal immune defenses. Given the complexity of VDAC1 interactions, future research needs to further explore how VDAC1 oligomers work in concert with other MOM proteins, such as the Bcl-2 family proteins BAK and BAX.

Blocking the inflammatory pathway driven by cytosolic mtDNA leakage, as an innovative therapeutic strategy targeting a core pathological mechanism, shows great potential in treating age-related diseases characterized by sterile inflammation. However, the clinical translation of this strategy must be carefully weighed against the risk of impairing the host’s normal immune defenses. Therefore, future research should focus on developing more targeted, time-controlled drug delivery systems and identifying reliable biomarkers to guide precise clinical application.

### 4.2 Inhibition of the cGAS-STING signaling pathway

In age-related diseases, intervention strategies targeting the blockade of cGAS or STING proteins have become a significant research direction for delaying senescence phenotypes, alleviating chronic inflammation, and improving organ function. First are small molecule inhibitors. In naturally aging mouse models, the STING inhibitor H-151 significantly improved cardiac function and reduced cardiac inflammation and cellular senescence phenotypes (94). In renal tubular epithelial cells (HKC-8), H-151 attenuated senescence-induced SASP expression (65). In terms of targeted delivery strategies, researchers have developed nanoparticles encapsulating cGAS/STING inhibitors (SPINs) to achieve targeted delivery and sustained release. Degradable nanocarriers were constructed using PLGA (polylactic-co-glycolic acid) to load the cGAS inhibitor RU.521 and the STING inhibitor H-151 respectively, and a polymer with strong π-π affinity, P(HPMA-Bz), was blended into the PLGA matrix to significantly enhance drug loading and modulate release kinetics. The nanoparticles are phagocytosed by macrophages and slowly release the inhibitors intracellularly, inhibiting cGAS-cGAMP synthesis or STING-palmitoylation to reduce Type I IFN and block M1 polarization. This strategy may also be effective for neurodegenerative diseases such as Alzheimer’s, Parkinson’s, and Huntington’s disease, holding significant clinical translation potential (95). Nevertheless, multiple translational bottlenecks limit the *in vivo* application of such nanodelivery platforms: first, the blood-brain barrier severely restricts the cerebral accumulation of PLGA-based nanoparticles for central nervous system aging disorders; second, non-specific phagocytosis of nanoparticles by systemic immune cells leads to off-target STING suppression, which may impair physiological anti-pathogen immune surveillance and tumor immune monitoring; third, long-term biosafety of degradable polymer nanocarriers in aged individuals with multi-organ hypofunction remains insufficiently validated by long-term toxicology studies. Future research needs to develop tissue-targeted surface-modified nanoparticles to reduce systemic immunosuppressive side effects. The main advantages of nanodelivery systems are their ability to achieve targeted or localized drug release, thereby avoiding the risks of infection and cancer associated with systemic immunosuppression (STING-Pathway Inhibiting Nanoparticles (SPINs) as a Platform for Treatment of Inflammatory Diseases.), and improving drug stability, selective uptake, and tissue-specific enrichment (96). However, clinical translation still faces challenges, including the efficiency of drug crossing the blood-brain barrier, off-target effects, and potential immunosuppressive risks (97).

In addition to small molecule inhibitors, some natural bioactive components have also been shown to regulate the cGAS-STING signaling pathway. In aging mouse models, inhibiting the cGAS-STING pathway can improve cognitive dysfunction, and reduce neuroinflammation and oxidative stress. The natural compound Punicalin has been shown to exert anti-brain aging effects by inhibiting this pathway (98). Punicalin is a high-molecular-weight ellagitannin featuring a unique macrocyclic structure. It consists of a glucose core bonded to two gallagic acid units, which are in turn derived from the oxidative coupling of two gallic acid moieties and two hexahydroxydiphenoyl (HHDP) groups (99). Mechanistically, punicalin not only directly scavenges free radicals but also activates the Nrf2-HO-1 antioxidant signaling axis and inhibits key pro-inflammatory pathways such as NF-κB, MAPK, and cGAS-STING, thereby significantly reducing oxidative stress and inflammation in cells and tissues. In the nervous system, its protective effects are manifested as inhibiting the overactivation of microglia and astrocytes, reducing neuronal apoptosis, restoring synaptic plasticity, and improving cholinergic function. Preclinical evidence from D-galactose-induced accelerated aging mouse models and various inflammation/oxidative stress models shows that punicalin improves cognitive function, exerts anti-inflammatory and antioxidant effects, and promotes cerebral blood flow in a dose-dependent manner. Its acute and chronic toxicology data and pharmacokinetic profiles also demonstrate good safety, supporting the feasibility of advancing this compound into Phase I/II clinical trials. Mild Cognitive Impairment (MCI) and early AD are the primary target indications. Meanwhile, oral formulation technologies such as microencapsulation or nanodelivery systems are being employed to enhance its bioavailability. If successful, this research will provide a multi-target intervention strategy derived from a natural product for the growing aging population, filling a gap in the treatment of neurodegenerative diseases with current anti-inflammatory and antioxidant drugs. Future research should further elucidate the direct interaction patterns between punicalin and the cGAS and STING proteins, and systematically evaluate the specific contribution of its major metabolites (especially urolithin A) in inhibiting the cGAS-STING pathway to determine whether the parent molecule or its metabolites play the leading role in clinical development. Resveratrol has been shown to mitigate microglial senescence and cognitive decline by inhibiting the translocation of STING from the endoplasmic reticulum to the Golgi apparatus and the downstream phosphorylation of TBK1 (100). The advantages of resveratrol are its ease of synthesis and administration, while its disadvantages may include limited specificity and potency, as well as potential issues with *in vivo* metabolism and bioavailability of a natural product (101).

In summary, targeting cGAS-STING, a core driver of aging, opens up a new track for the development of senotherapeutics. However, a major challenge remains in how to distinguish and target harmful, pro-pathological senescent cells while preserving those that play beneficial roles in tissue repair and tumor suppression. Additionally, the long-term effects of inhibiting the cGAS-STING pathway on the immune system, tumor surveillance, and overall health are not fully understood and require long-term preclinical and clinical studies for evaluation (**Table 1**).

**Table 1:** mtDNA-cGAS-STING axis inhibitors.

Target	Inhibitor	Mechanism	Application cell and animal	Disease	Reference
STING	H151	H-151 severs the signaling pathway from cGAS-STING to downstream inflammatory and interferon responses by covalently modifying the Cys91 site of STING	Mov10-cKO mice, HEK293T cells	Age-related cardiac dysfunction	(85)
	C-176	STING inhibitors, blocking the cGAS-STING pathway, targeting the upstream mechanism of type I interferon production triggered by cytosolic DNA sensing	Primary human bronchial epithelial cells	COPD	(92)
Sirt3	Honokiol	By activating SIRT3, HKL promotes the deacetylation of SOD2 and OGG1 to alleviate mitochondrial oxidative stress and mtDNA damage, and inhibits the cGAS-STING-NF-κB pathway	A549 cells;mouse model of silicosis	Silicosis, Aging	(83)
SARM1	D-Pinitol	By blocking SARM1-mediated NAD+ depletion, DP effectively restored mitochondrial function and reduced cytosolic mtDNA leakage, in turn inhibiting the downstream cGAS-STING inflammatory pathway and the release of SASP	Mouse model of renal failure	Renal failure	(93)
VDAC1	VBIT-4	Inhibit macropore formation on the MOM, blocking the cGAS-STING pathway	5 × FAD mice	Alzheimer’s disease	(94)
mtDNA	EQ NPs	Reduce the generation of Ox-mtDNA, prevent mPTP opening, adsorb cytosolic mtDNA, and activate mitophagy	Diabetic mouse model	Diabetes mellitus	(84)
cGAS-STING	LNT-PBAE-G-ND	Activate the cGAS-STING signaling pathway to induce macrophage activation and immune enhancement	Murine Macrophages	AD, PD	(86)
	Punicalin	Inhibition of cGAS-STING pathway activation blocks the downstream phosphorylation of TBK1/p65/IRF3, thereby effectively downregulating the SASP	Aging mouse model	AD	(88)
	Urocanic acid	Inhibition of the cGAS-STING inflammatory pathway in astrocytes by directly targeting the protein ZCCHC3 significantly alleviates D-galactose-induced cognitive impairment and neuroaging	Primary Mouse Astrocytes	Neurodegenerative Diseases	(95)

## 5 Conclusions and prospects

In summary, the mtDNA-cGAS-STING axis plays a critical role in inflammation regulation across various tissues. Distinct organs possess unique mtDNA release thresholds and differential signal integration patterns, which collectively form organ-specific “immune fingerprints,” providing a key perspective for understanding the heterogeneity of “inflamm-aging” at the organ level. Moving forward, research in this field must transition from early observational descriptions to the granular deconstruction of molecular mechanisms. Specifically, the deployment of cutting-edge technologies, such as single-cell multi-omics, spatial transcriptomics, and organ-on-a-chip models, we can not only precisely map the topology of mtDNA leakage and local immune responses but also reconstruct their true evolutionary processes within specific pathological microenvironments. This paradigm shift from macroscopic association to microscopic resolution is poised to overcome the limitations of current therapeutic strategies, facilitating the development of organ-specific “seno-metabolic” interventions and providing a new theoretical foundation for the precision medical management of age-related systemic inflammation.
